# Large-scale prediction of protein ubiquitination sites using a multimodal deep architecture

**DOI:** 10.1186/s12918-018-0628-0

**Published:** 2018-11-22

**Authors:** Fei He, Rui Wang, Jiagen Li, Lingling Bao, Dong Xu, Xiaowei Zhao

**Affiliations:** 1 School of Information Science and Technology, Northeast Normal University, Changchun, 130117 China; 2 Institution of Computational Biology, Northeast Normal University, Changchun, 130117 China; 3 Laboratory of Symbolic Computation and Knowledge Engineering of Ministry of Education, Jilin University, Changchun, 130012 People’s Republic of China; 4 Department of Electrical Engineering and Computer Science Christopher S. Bond Life Sciences Center, University of Missouri, Columbia, MO 65211 USA

**Keywords:** Protein ubiquitination site, Multiple modalities, Deep learning, Convolution neural network, Deep neural network

## Abstract

**Background:**

Ubiquitination, which is also called “lysine ubiquitination”, occurs when an ubiquitin is attached to lysine (K) residues in targeting proteins. As one of the most important post translational modifications (PTMs), it plays the significant role not only in protein degradation, but also in other cellular functions. Thus, systematic anatomy of the ubiquitination proteome is an appealing and challenging research topic. The existing methods for identifying protein ubiquitination sites can be divided into two kinds: mass spectrometry and computational methods. Mass spectrometry-based experimental methods can discover ubiquitination sites from eukaryotes, but are time-consuming and expensive. Therefore, it is priority to develop computational approaches that can effectively and accurately identify protein ubiquitination sites.

**Results:**

The existing computational methods usually require feature engineering, which may lead to redundancy and biased representations. While deep learning is able to excavate underlying characteristics from large-scale training data via multiple-layer networks and non-linear mapping operations. In this paper, we proposed a deep architecture within multiple modalities to identify the ubiquitination sites. First, according to prior knowledge and biological knowledge, we encoded protein sequence fragments around candidate ubiquitination sites into three modalities, namely raw protein sequence fragments, physico-chemical properties and sequence profiles, and designed different deep network layers to extract the hidden representations from them. Then, the generative deep representations corresponding to three modalities were merged to build the final model. We performed our algorithm on the available largest scale protein ubiquitination sites database PLMD, and achieved 66.4% specificity, 66.7% sensitivity, 66.43% accuracy, and 0.221 MCC value. A number of comparative experiments also indicated that our multimodal deep architecture outperformed several popular protein ubiquitination site prediction tools.

**Conclusion:**

The results of comparative experiments validated the effectiveness of our deep network and also displayed that our method outperformed several popular protein ubiquitination site prediction tools. The source codes of our proposed method are available at https://github.com/jiagenlee/deepUbiquitylation.

## Background

Ubiquitin is discovered by Goldstein et al. [1] in 1975, which is a small protein consists of 76 amino acids [2]. Under the effects of E1 activation, E2 conjugation and E3 ligation enzymes, ubiquitin may conjugate to a substrate protein on a certain lysine residue [3, 4]. ubiquitination is one of the most important reversible protein posttranslational modifications (PTMs) and plays the significant roles in protein degradation and other cellular functions [5, 6]. The ubiquitination system is also associated with immune response, cellular transformation and inflammatory response [7].

Owing to its importance and complexity of ubiquitination, recognizing potential ubiquitination sites contributes to obtaining a deep understanding of protein regulation and molecular mechanism. Traditional experimental techniques such as CHIP-CHIP analysis and mass spectrometry are time-consuming, costly and laborious, while computational approaches that could effectively and accurately identify protein ubiquitination sites are urgently needed.

Some computational methods have been developed for the identification of protein ubiquitination sites. Huang et al. [8] developed a predictor called UbiSite, which fused multiple features such as amino acid composition (AAC), positional weighted matrix (PWM), position-specific scoring matrix (PSSM), solvent-accessible surface area (SASA) and MDDLogo-identified substrate motifs into a two-layer Support Vector Machine (SVM) model to predict protein ubiquitination sites. Nguyen et al. [9] also applied SVM to build the prediction model, using three features including amino acid composition, evolutionary information and amino acid pair composition. Additionally, the motif discovery tool, MDDLogo, was also used in their predictor. Qiu et al. established the tool iUbiq-Lys [10], which adopted sequence evolutionary information and gray system model, to identify protein ubiquitination sites. Chen constructedUbiProber [11] to combine sequence information, physico-chemical properties and amino acid composition with SVM, In which they respectively trained general model for a eukaryotic proteome and species-specific model for three species-specific proteomes. ESA-UbiSite [12] proposed by Wang et al., introduced physico-chemical properties into SVM. But they applied evolutionary screening algorithm (ESA) to select effective negative dataset from the whole dataset.

These existing machine learning approaches have good performance on small-scale data, nevertheless, there are still some challenges for large-scale protein ubiquitination site prediction: (1) Weakness of artificially designed features. All existing methods utilized feature engineering in feature extraction stage, which relied on expert knowledge, and usually lead to incomplete and biased feature vectors [13, 14]. (2) Heterogeneity among features. Almost existing prediction tools chose to fuse multiple features to improve the accuracy, but neglected the intrinsic heterogeneity among them. (3) Unbalanced distributions between positive and negative samples [15]. In the whole proteome, only a small part of lysine residues can be attached to ubiquitin, which determines protein ubiquitination site prediction as an extreme unbalanced issue. Existing methods do not perform well in identifying potential protein ubiquitination site under such unbalanced circumstance. Deep learning as a trendy machine learning technique for large scale data is considered promising to solve these problems. It provides multiple-layer networks and non-linear mapping operations to excavate deep characteristics and reveal their internal association, especially on large-scale data. The deep-learning framework detects potential complex patterns from raw input signals, and generates homogenous deep representations for classification tasks. A variety of deep learning networks have been applied to genomic and proteomic analyses successfully [16–18]. However, deep learning technique is yet to utilize to predict protein ubiquitination sites.

In this paper, we established a multimodal deep architecture by using three different kinds of protein modalities, namely raw protein sequence fragments, selected physico-chemical properties of amino acids, and corresponding position-specific scoring matrix (PSSM). In the deep architecture, we built multiple convolution layers for detecting raw information from protein sequence representations, and combined the physico-chemical properties of amino acids with the help of some stacked fully connected layers, and brought other multiple convolution layers to explore the evolutionary profile toward potential ubiquitination sites. Then, such three sub-nets were trained separately so that these multiple modalities were transformed into more compatible representations for combination to predict unseen protein ubiquitination sites. As far as we know, this is the first published work that employs deep architecture to protein ubiquitination site prediction.

## Methods

### Large scale dataset collection

For implementing the large scale prediction of ubiquitination sites, we collected data from Protein Lysine Modification Database 3.0 version (PLMD) consisting of 25,103 proteins with 121,742 ubiquitination sites. PLMD is a specialized dataset containing 20 types of protein lysine modifications, and extends from CPLA 1.0 dataset and CPLM 2.0 dataset. As we know up to now, this is probably the largest-scale available protein ubiquitination database, and is never referred in any other researches of protein ubiquitination site prediction. For the sake of avoiding overestimation caused by homologous sequences, we utilized CD-HIT tool [19] to screen the similar protein sequences by 40% similarity in all data, and finally extracted 60,879 annotated protein ubiquitination sites from 17,406 proteins. Moreover, these protein sequences were divided into training dataset and testing dataset by random partition for constructing prediction model. Thus, there are totally 12,100 protein sequences with 54,586 ubiquitylated sites in training dataset and 1345 proteins with 6293 ubiquitylated sites in the independent testing dataset.

We used a conventional way to segment protein fragments with central lysine residues and fixed window size of 2*n* + 1, in which *n* was the number of upstream or downstream flanking amino acids around the targeting lysine residue. Furthermore, to control the interference that some negative training samples may be homologous to positive training samples, the tool CDHIT- 2D was utilized to remove the negative samples with 50% similarity to positive samples [8]. For building unbiased models, a relative small proportion of 30% from training samples were extracted as validation samples by random sampling in every epoch of training process. The details of experimental datasets are shown in Table 1.

**Table 1:** Details of training dataset, validation dataset and independent testing dataset

Data set	Description			
	Number of sequences	Number of positive data	Number of negative data	Note
Training	12,100	7733	250,054	Random partitioning in each training iteration
Validation		1547	50,010	
Testing	1345	6293	46,080	Reservation

### Encoding of protein fragments

In this paper, we employed three types of encoding schemes to represent the protein sequence fragments.

1. One hot vector: every sample included *m* amino acids was constructed as an *m* × *k* 2-dimensional (2D) matrix, using a *k* dimensional zero vector with a one corresponding to the amino acid at the index of protein sequence. We assigned 0.05 to the positions whose left or right neighboring amino acids cannot fit the window size. Therefore, each protein fragment was mapped into a sparse and exclusive coding within its relative position information.
2. Physico-chemical properties: Prior researches [15, 20] demonstrated that there were strong correlations between physico-chemical properties of amino acids and ubiquitination sites. Many researches introduced physico-chemical properties in diverse protein post-translation modification site predictions such as acetylation, phosphorylation and sulfation [11]. These physico-chemical properties corresponding to each amino acid can be found in an AAindex database [21]. It recorded 544 physico-chemical properties which would lead to excessive model parameters in deep architecture [22]. To reduce redundancy information and control complexity of model, we only select top thirteen physico-chemical properties that have been validated strongly related to ubiquitination in literature [11], and then a *m* × 13 2D matrix was formulated as another encoding modality for each sample. The details of these selected physio-chemical properties are shown in Table 2.
3. PSSM Profile: In this paper, we also employed PSSM to represent the evolutionary profile of the protein sequence. We referred the non-redundant database Swiss-Prot as the search source, generating the raw PSSMs of all protein sequences by utilizing the Basic Local Alignment Search Tool (BLAST) with the parameter “-*j* 3 -*h* 0.001” [23]. In one raw PSSM, a 20 dimensional vector demonstrated approximately the preference of 20 types of amino acids at each position of protein sequence. In order to focus on the potential ubiquitination sites, we extracted the PSSM fragment corresponding to the window size *m* from the PSSM matrix from the whole protein sequence, which recorded the position-specific evolutionary profiles of protein fragment. Hence, we obtained an *m* × 20 2D matrix as PSSM encoding for each protein fragment.

**Table 2:** The selected physico-chemical properties

Physico-chemical property	Description
EISD860102	Atom-based hydrophobic moment
ZIMJ680104	Isoelectric point
HUTJ700103	Entropy of formation
KARP850103	Flexibility parameter for two rigid neighbors
JANJ780101	Average accessible surface area
FAUJ880111	Positive charge
GUYH850104	Apparent partition energies calculated from Janin index
JANJ780103	Percentage of exposed residues
JANJ790102	Transfer free energy
PONP800102	Average gain in surrounding hydrophobicity
CORJ870101	NNEIG index
VINM940101	Normalized flexibility parameters, average
OOBM770101	Average non-bonded energy per atom

### Multimodal deep architecture construction

**Fig. 1:**
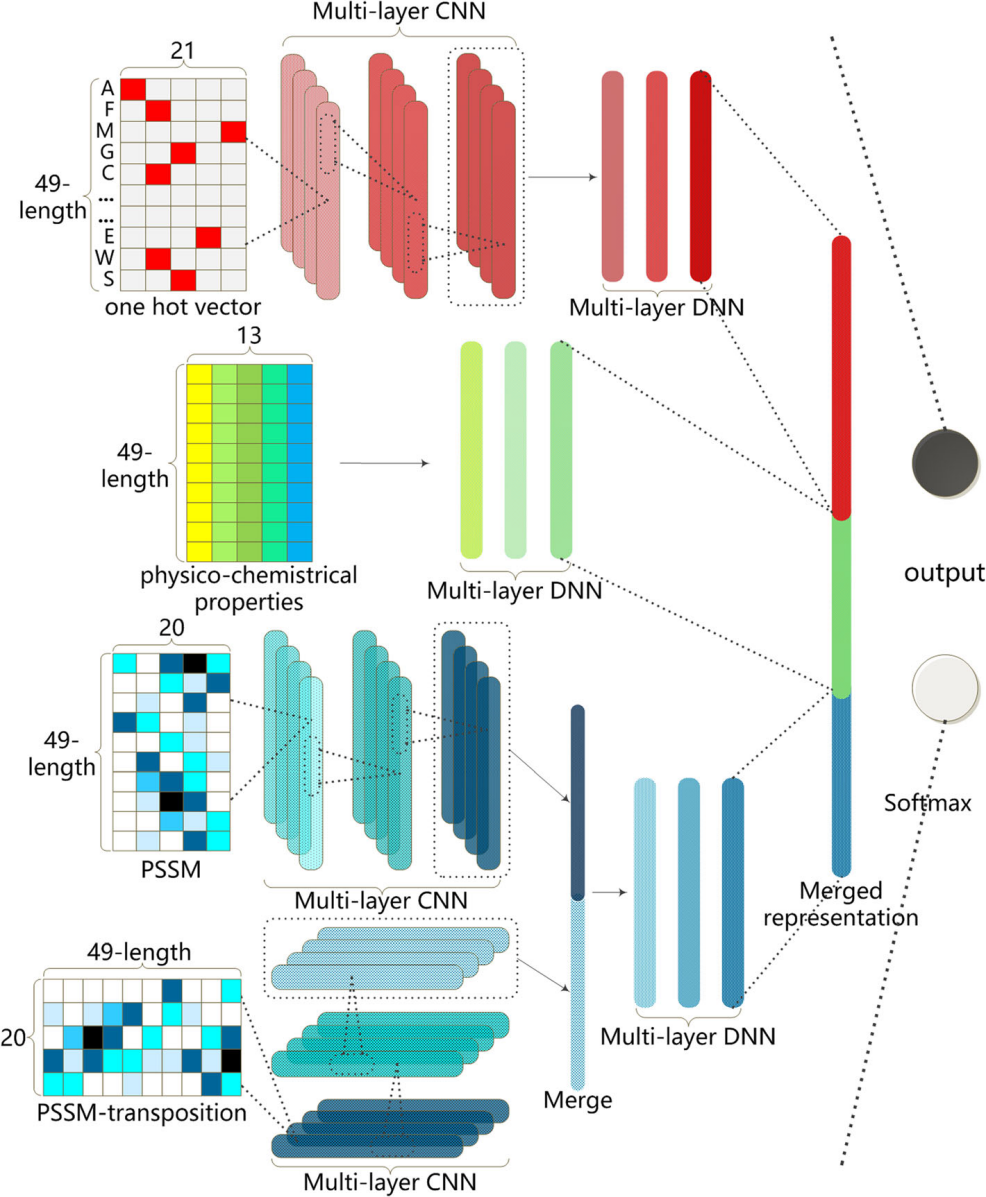
The structure of the proposed deep architecture

As Fig. 1 shown, we could find that our deep architecture includes three parts of sub-nets dealing separately with the above three kinds of input feature encodings. After that, we chose to merge their output layers for combining the three modalities [24].

For the purpose of precisely detecting implicit sequence-type features, we used 3 hidden layers of one dimensional Convolution Neural Network (1D CNN) to process one hot vector. Because of its inherent sparsity [25], a main function of CNN is to transform one hot vector into a given range of feature maps as detected sequential information. When this hierarchical convolution process ended, all newly generated feature maps were merged together into three fully connected dense layers, which may produce lower dimensional feature representations [26]. We found that this structure was impactful to detect sequential feature representations.

For physico-chemical properties, a Deep Neural Network (DNN) with three dense layers was introduced to generate their deep representations [27]. Physico-chemical properties reflected characteristic of proteins from various prospective, so that fully connected DNN structure that interconnects all these factors was utilized for their joint effect and useful combination. .

For the input modality of PSSM, we mainly applied 1D CNN with 3 hidden layers to detect potential informative descriptions among amino acids through evolution to the protein fragment. Differing from the sub-net of one hot vector, the trans-positioned PSSM vector was inputted into another three layers 1D CNN to obtain deep evolutionary characterization among different sequence positions. Then the feature maps involving two 1D CNNs were jointly merged to produce completely PSSM representations by three following fully connected dense layers.

Next, the output states of three sub-nets are merged into a mixed representation for fusing the three deep representations of multiple input modalities at higher level, where the mutual heterogeneity among their raw shallow representations was eliminated. This part of structure composed of dense layers and a 2-state output layer for implementing binary classification activating by softmax function. The weights between merged layer and output layer may be regarded as the contributions from three input modalities. All hyper-parameters of our deep architecture are detailed in Table 3.

**Table 3:** The hyper-parameters of the proposed deep architecture

Subnet	Layer	Hyper-parameters			
		Activation function	Size^*c*^	Filters	Drop-out
One hot vector	1D Convolution	softsign	2	200	0.4
		softsign	3	150	0.4
		softsign	5	150	0.4
		softsign	7	100	0.4
	Dense^a^	relu	256	–	0.3
		relu	128	–	0
		relu	128	–	–
Phsico- chemical properties	Dense	softplus	1024	–	0.2
		softplus	512	–	0.4
		softplus	256	–	0.5
		relu	128	–	–
PSSM profile	1D Convolution	relu	1	200	0.5
		relu	8	150	0.5
		relu	9	200	0.5
	1D Convolution^b^	relu	1	200	0.5
		relu	3	150	0.5
		relu	7	200	0.5
	Dense	relu	128	–	0.3
		relu	128	–	0
Merged representations	Dense	softmax	2	–	0

^a^Dense layers represent for the fully connected layers in keras

^b^The layers were designed for trans-positioned PSSM profile

^c^The size of convolution layers means the kernel sizes, and the size of Dense layers denotes the number of hidden states

For controlling the training process under balanced data, one training strategy was introduced to our model. Considering the considerable model parameters in three subnets, each subnet was respectively trained to guarantee the optimality of their weights, and then reloaded these trained weights as initialization to the whole multi-modal deep architecture. In the following training process of whole network, overall weights including the weights of last merged layer would be fine-tuned until they achieved global optimum. We implemented the training procedure of the whole deep architecture and subnets following the bootstrapping strategy. Let *pos* and *neg* represented the number of positive samples and negative samples respectively. Because of relative small size of positive samples, *pos* negative samples were randomly chosen to build balanced training dataset with all positive samples in each bootstrapping iteration [28, 29]. Therefore, all negative samples were divided into *N* = ⌊*neg*/*pos*⌋ bins, and our deep-learning network would be trained *N* times. The early stop rule [30] was introduced to control epoch numbers in our work, and the training process stopped automatically by the time the observed metric had not changed any more for a default epoch iterations (50 in this study).

We established this deep architecture using Keras 1.1.0 with Theano 0.9, and ran it on a graphic processing unit (GPU) GTX1080Ti. Due to the advantage of GPU computations and no need of feature engineering in modeling, the average time for predicting ubiquitination sites in a protein was in a few minutes, although it took about 2 h to train the model on 12,100 protein sequences. Nevertheless, the training process only needed to conduct once.

## Result and discussion

### Performance of the multimodal deep architecture

**Fig. 2:**
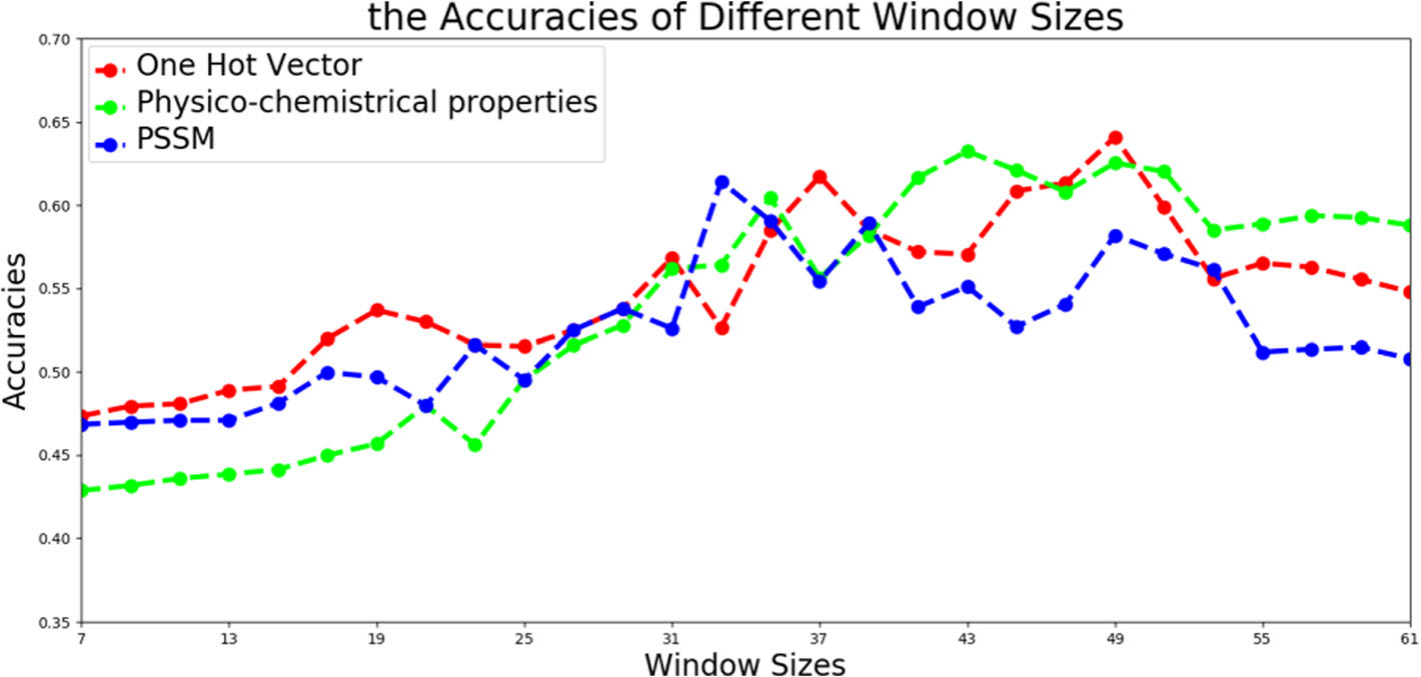
The accuracies of validation samples using different window sizes on three modalities

Window size for cutting protein fragments is an important variable for protein ubiquitination sites [31], owing to its direct effect on the representation and information involving in modeling. We designed experiments to search for feasible values of window size for our deep architecture. The attempts started with the window length 7 and ended at the window length 61(*n* was from 3 to 30). For each candidate, protein sequence fragments cutting from protein sequence were encoded into three types of input modality, to train corresponding deep network one by one. We displayed the performance of different window sizes using one hot vector, physico-chemical properties and PSSM profile on the validation samples in Fig. 2.

In Fig. 2, we can see that when window size reached to 49, the three kinds of modalities achieved comparable accuracies to other candidates. This conclusion was inconsistent with some existing studies [8, 11], which implied that our deep architecture needed longer sequence fragments to offer potential long distance information.

**Fig. 3:**
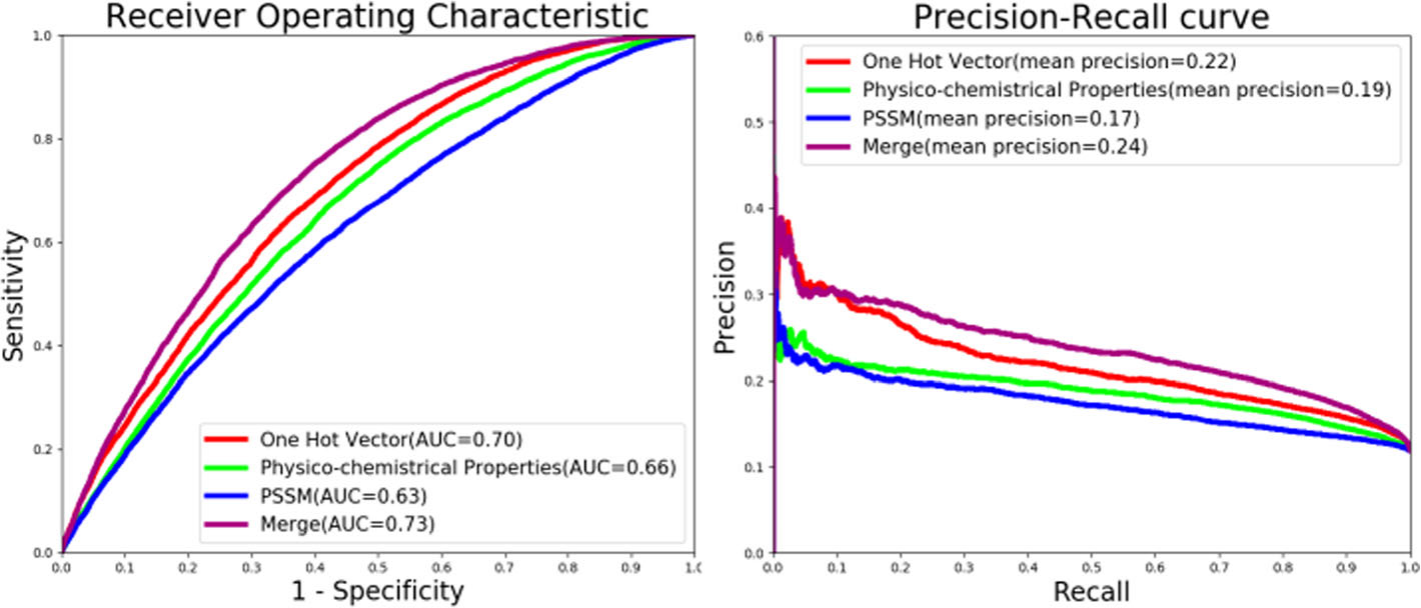
ROC and precision-recall curves comparing our multi-modal network and subnets of uni-modality

Subsequently, we trained three subnets using the three modalities including one hot vector, physico-chemical properties and PSSM profile. The generative ROC (receiver operating characteristic) curves and precision-recall curves of uni-modal subnets and multi-modal deep network were plotted in Fig. 3.

Benefiting from the data-driven combination way, the whole multi-modal network achieved better performance than any subnets of uni-modality. The AUC (area under the ROC curves) and mean precision (area under the precision-recall curves) of multi-modal deep network reached 0.73 and 0.24 as shown in Fig. 3. Due to the pre-training of three subnets, the optimal weights of trained subnets for one hot vector, physico-chemical property and PSSM profile would be searched in advance for combination. Thus, the applicable weights of whole multi-modal deep architecture was able to appear by the following supervised fine tune. Figure 3 also indicated that one hot vector outperformed among three input modalities. It suggested that deep learning architecture may detect effective potential features hidden in raw protein sequences.

**Fig. 4:**
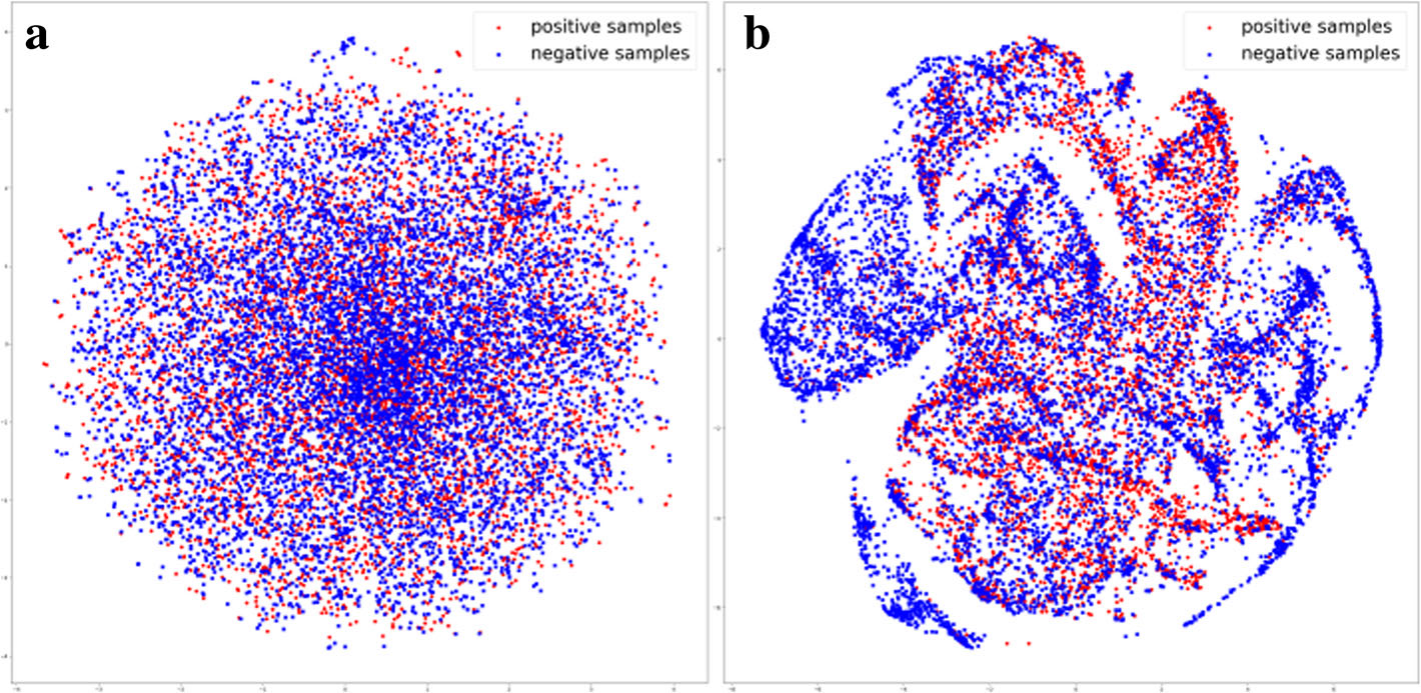
t-SNE visualization of (**a**) input layers and (**b**) merged layer

In order to exhibit the rationality and validity of our deep architecture, the discrimination among testing samples was plotted in 2D coordinate using t-SNE [32] as Fig. 4 shown. It obviously showed that positive samples and negative samples tended to be separated after multilayer processing, which implied that our multi-modal deep architecture may detect distinguishing representations from three different modalities and fuse them to further enhance their discriminative ability.

### Comparisons with other classifiers

In the next stage, we would like to compare our deep architecture with two most popular used protein ubiquitination site prediction classifiers: SVM and Random Forest. For fair comparisons, all three types of input modalities, namely one hot vector, physico-chemical properties, and PSSM profile were used to train SVM model and Random Forest model severally. In addition, all the three modalities were also concatenated into a vector called merged feature, which was sent to train another model independently. Considering the unbalanced training samples, we randomly extracted the same number of positive samples and negative samples to form training data in each training process. All these models were trained with 10-fold cross-validation using the same experimental protocol. Their results were combined with those of our deep architecture in Table 4.

**Table 4:** Comparative results with SVM classifier and Random Forest

Model	Input	Metrics			
		Accuracy	Sensitivity	Specificity	MCC
SVM	One hot vector	59.65%	46.69%	61.42%	0.054
	Physico-chemical property	57.36%	43.84%	59.22%	0.051
	PSSM	55.71%	44.29%	57.84%	0.047
	Merged	56.92%	44.34%	58.97%	0.049
Random Forest	One hot vector	57.27%	45.01%	58.94%	0.026
	Physico-chemical property	56.55%	47.40%	57.80%	0.034
	PSSM	54.19%	44.98%	56.32%	0.021
	Merged	56.52%	46.36%	58.83%	0.024
Our deep architecture	One hot vector	64.15%	64.41%	64.08%	0.189
	Physico-chemical property	61.84%	60.97%	61.95%	0.151
	PSSM	56.82%	58.73%	56.57%	0.099
	Merged	66.43%	66.67%	66.40%	0.221

Table 4 indicated that our deep architecture was superior to other models. The SVM and random forest models using uni-modal obtained general high specificity and a low sensitivity. It can be concluded that these traditional machine learning modeling approaches were incapacity of generating discriminative features from raw inputs. That is the reason why existing tools did not choose to input raw sequence fragments and properties, while further transformed these modalities into meaningful feature vectors, i.e. amino acid composition, for modeling. Meanwhile our deep architecture had the ability of detecting useful information from raw sequence fragments without feature engineering. The same situation occurred in the experiments of multi-modalities among different, which revealed that our deep architecture may carry out multi-modal fusion in a conductive way. The overall estimator Matthews correlation coefficients (MCC) of the traditional machine learning models were much lower than that of our architecture, which reflected that our bootstrapping training strategy may consolidate the generalization of our architecture on unbalanced training dataset from another respective.

### Comparisons with other protein Ubiquitylation site prediction tools

We compared our proposed with several popular protein ubiquitination site prediction tools namely Ubisite [8], iUbiq-Lys [10], UbiProber [11], and ESA-UbiSite [12] by submitting our testing dataset to their websites. The assessed metrics were calculated according to the results from these websites in Table 5.

**Table 5:** Comparison of independent testing performance with other ubiquitination site prediction tools

Tool	Metrics			
	Accuracy	Sensitivity	Specificity	MCC
ESA-Ubisite	61.26%	46.14%	63.34%	0.064
UbiProber	55.06%	62.40%	54.05%	0.107
iUbiq-Lys	84.63%	3.35%	96.88%	0.005
Ubisite	73.63%	29.62%	79.64%	0.073
Our deep architecture	66.43%	66.67%	66.40%	0.221

**Fig. 5:**
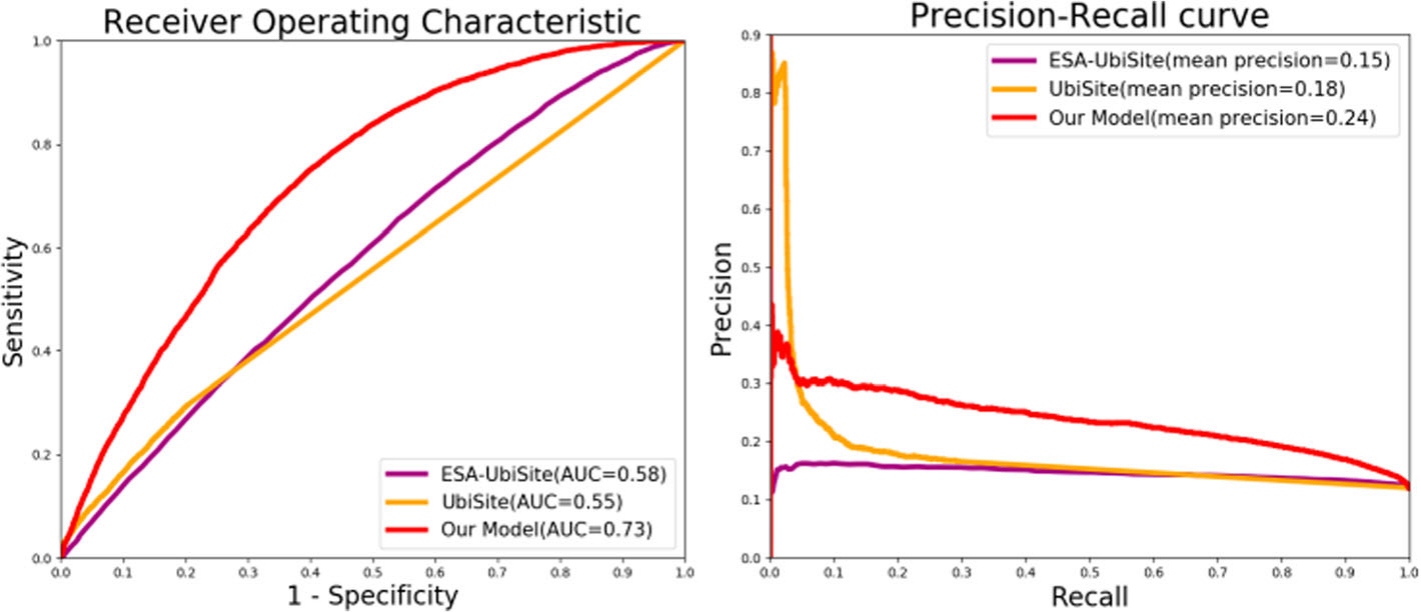
The ROC and precision-recall curves comparing proposed deep architecture and other protein ubiquitination site prediction tools

From Table 5, it can be found that our deep architecture yielded an acceptable performance, including 66.43% accuracy, 66.7% sensitivity, 66.4% specificity, and 0.221 MCC value with a 0.5 decision threshold. With regard to the unbalanced negative distribution of testing samples, our deep architecture showed unbiased prediction results in terms of equilibrium sensitivity and specificity. Our highest sensitivity among all tools demonstrated that our deep architecture can identify potential protein ubiquitination sites more effectively. Moreover, we plotted the ROC and precision-recall curves with AUC and mean precision of Ubisite, ESA-Ubisite and our model in Fig. 5. The reason why the ROC of other tools were absent in Fig. 5 was that these websites only returned predicted decisions instead of predicted scores.

Figure 5 exhibited that our model had evident overall advantages in terms of ROC and precision-recall carves. It proved high confidence of deep architectureon large-scale protein ubiquitination site data. It is worth noting that under a certain minor recall, Ubisite achieved higher precision among the three methods,probably because Ubisite introduced more prior knowledge from positive training samples to its classification model. It divided positive training samples into 12 subgroups according to the clustered results of significant substrate motifs using the MDDLogo tool [33]. And then it trained 12 sub-models using the 12 subgroups of positive training samples and the same number of negative samples to implement a boosting classification. Such classification models emphasized the feature patterns of positive samples, and guided to detect potential homologous protein fragments with high similarity to its positive training samples. Consequently, it resulted in better precision than that of our deep architecture only when the recall was less than 3.89%.

Furthermore, a series of extensive comparative experiments were carried out on independent testing datasets. We tried our best to collect the data sets used in the above mentioned three literatures. CPLM [34], UniprotKB/Swiss-Prot, hCKSAAP [35]. However, due to data release upgrade, and random division in their experiments, we cannot reappear the identically experimental data in the literatures. By inputting these collective datasets into our deep architecture, we can observe their prediction results as shown in Table 6. Our deep architecture also performed effectively and powerfully on these collective datasets.

**Table 6:** The performance of our deep architecture on different datasets

Datasets	Metrics			
	Accuracy	Sensitivity	Specificity	MCC
CPLM	74.39%	60.44%	74.72%	0.120
Swiss-prot	64.28%	68.95%	63.81%	0.193
hCKSAAP	73.97%	73.76%	73.98%	0.26
PLMD	66.43%	66.67%	66.40%	0.221

Even though our deep learning architecture promoted the performance of protein ubiquitination site prediction on large scale data, there is still room for improvement. In the future, we would like to continue studying the optimization strategyfor guiding the selection of deep learning hyper-parameters, and cooperate with biologists to upgrade the model more biologically interpretable and reliable.

## Conclusion

In this paper, a multimodal deep architecture was proposed method to predict large scale protein ubiquitination sites. Three different modalities include one hot vector, physico-chemical properties and PSSM, were employed to build the predition model. Comparative results on the available largest scale protein ubiquitination site database PLMD validated the effectiveness of our method. From the t-SNE visualization, it can be found that our deep architecture can generate powerful discriminative features to distinguish ubiquitination sites from non-ubiquitination sites in protein sequences. The success of our method is mainly due to the data-driven feature detection in deep learning, the multimodal fusion of deep representations, and the bootstrapping algorithm. Our source codes are freely available at https://github.com/jiagenlee/deepUbiquitylation.
